# Nanotribological and Nanomechanical Properties Changes of Tooth After Bleaching and Remineralization in Wet Environment

**DOI:** 10.1186/s11671-015-1167-x

**Published:** 2015-12-01

**Authors:** Dandan Yu, Shanshan Gao, Jie Min, Qianqian Zhang, Shuai Gao, Haiyang Yu

**Affiliations:** State Key Laboratory of Oral Diseases, West China Hospital of Stomatology, Sichuan University, Chengdu, China

**Keywords:** Dental bleaching, Nanoscratch, Nanoindentation, Tooth enamel

## Abstract

Teeth bleaching cases had increased with people’s desire for oral aesthetic; however, bleached teeth would still undertake chewing actions and remineralizing process in saliva. Nanotribological and nanomechanical properties are proper displays for dental performance of bleached teeth. The purpose of the research was to reveal the effect of bleaching and remineralization on the nanotribological and nanomechanical properties of teeth in wet environment. The specimens were divided into four groups according to the bleaching products used: 12 % hydrogen peroxide (HP) (12HP group); 15 % carbamide peroxide (CP) (15CP group); 35 % CP (35CP group); and artificial saliva (control group). The nanotribological and nanomechanical property changes of tooth enamel after bleaching and remineralization were evaluated respectively by nanoscratch and nanoindentation tests in wet environment, imitating the wet oral environment. The morphology changes were evaluated by statistical parametric mapping (SPM) and scanning electron microscopy (SEM). After bleaching, 12HP group and 15CP group showed increased scratch depth with more pile ups on the scratch edges, decreased nanohardness, and corroded surface appearance. While the 35CP group showed an increase in nanoscratch depth, no change in nanohardness and surface appearance was observed. The control group showed no change in these measurements. After remineralization, the three bleaching groups showed decreased nanoscratch depth and no change of nanohardness compared with the bleached teeth. And the control group showed no changes in nanotribological and nanomechanical properties. The nanotribological and nanomechanical properties of the 12HP group and 15CP group were affected by bleaching, but the nanotribological properties recovered partly and the nanomechanical properties got no change after 1 week of remineralization. As for the 35CP group, the nanotribological properties were influenced and the nanomechanical properties were not affected. These results remind us of taking actions to protect our teeth during bleaching.

## Background

Dental bleaching could effectively improve teeth color and is receiving increasing popularity [1]. The active agent of bleaching products is hydrogen peroxide (HP) or carbamide peroxide (CP), which has a strong oxidizability and could react with the organic and inorganic substances in teeth [2]. The active agent could react with the black carbon ring structures and break them into unsaturated structure of light color [3, 4]. As the bleaching process went on, these unsaturated structures turned into colorless hydrophilic structures, and then the teeth became white [4, 5].

The oxidizability of HP or CP is nonspecific [6], so not only the target dark substances of teeth but also other normal organic or inorganic substances might be oxidized during bleaching [7]. Thus, these chemical components of teeth, closely related to nanotribological and nanomechanical properties, might be changed during bleaching as well, resulting in the different performance of nanotribological and nanomechanical properties [8]. Teeth undertake chewing actions every day, and excellent nanotribological and nanomechanical properties could guarantee teeth function.

However, seldom are there researches done on the nanotribological properties of teeth affected by bleaching [2, 9]. The only research about the nanotribological properties of bleached teeth was done in dry environment [2]. Chewing actions occur in wet oral environment, and previous researches showed that testing environment actually do affect the nanotribological properties [10]. Also, the change of nanotribological properties after remineralization of bleached teeth has not been reported. Therefore, the measurement of nanotribological and nanomechanical properties of normal natural teeth, bleached teeth, and remineralized teeth in oral-like environment could help assess the bleaching effect of tooth function.

Though there have been some researches on the change of teeth’s microhardness before and after bleaching, no defined conclusion were found [11, 12]. The reason might be that the test loads were too large, causing the test depth deeper than the influenced depth [13]. So we chose a nanohardness tester with the test load of 2000 μN for the indention test.

The purpose of the research was to explore the difference of nanotribological and nanomechanical properties between normal natural teeth, bleached teeth, and bleached-remineralized teeth in wet environment.

## Methods

The research was approved by the Research Ethics Committee, Sichuan University, China, and all teeth were collected with patients’ consent. Eighty freshly extracted premolars with no caries or cracks were used in the experiment (18 ~ 25 years old). They were stored in Hank’s balanced salt solution (HBSS) at 4 °C after cleaning. These teeth were cut at the cement-enamel junction and then cut along the tongue-labial axis. After that, all teeth blocks were embedded in self-curing epoxy resin (Struers, Copenhagen, Denmark) with the occlusal surface facing up. After totally curing of the resin, the occlusal surface was grounded flat with 800, 1200, 2400, and 4000 grit abrasive papers (Struers, Copenhagen, Denmark) under water irrigation and polished with alumina suspension slurry (Struers, Copenhagen, Denmark) of 3 μm for 5 min and OP-Nondry (Struers, Copenhagen, Denmark) of 0.04 μm for 10 min on a polishing machine (Struers, Copenhagen, Denmark). The resultant surface average roughness was less than 5 nm.

**Fig. 1:**
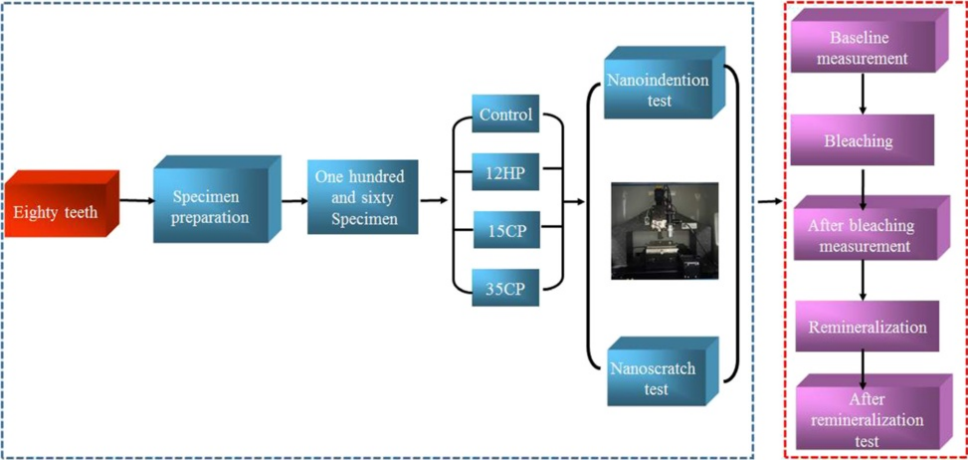
Specimen preparation, grouping, and the test process

Then, the specimens were randomly divided into four groups as shown in Fig. 1: 12HP group—Premium Dental Grade Teeth Whitening Gel (12 % HP; Premium Dental, USA); 15CP group—Opalescence PF (15 % CP; Ultradent Dental GmbH, Salt Lake City, USA); 35CP group—Opalescence PF (35 % CP; Ultradent Dental GmbH, Salt Lake City, USA); and control group—artificial saliva (AS), with 40 samples in each group. Information about the bleaching products was shown in Table 1.

**Table 1:** Bleaching products and the bleaching process in the research

Group	Products	Manufacturer	Active ingredients	Bleaching method	Application time per day
Control	AS	China	–	No bleaching	24 h in AS
12HP	Premium Dental Grade Teeth Whitening Gel	USA	12% HP	Apply bleaching products containing 12% HP on the samples under the lighting of a LED light	0.5 h bleaching and 23.5 h in AS
35CP	Opalescence PF	USA	35 % CP, 0.11 % (*w*/*w*) fluoride	Apply bleaching products containing 15% CP on the samples	6 h bleaching and 18 h in AS
15CP	Opalescence PF	USA	15 % CP, 0.11 % (*w*/*w*) fluoride	Apply bleaching products containing 35% CP on the samples	0.5 h bleaching and 23.5 h in AS

*HP* hydrogen peroxide, *CP* carbamide peroxide

The bleaching procedure was performed for 7 days according to manufacturer’s instructions as shown in Table 1. For each specimen, 0.04 ml bleaching agent was applied daily to the enamel surface. After bleaching, the gel was rinsed off with HBSS for 15 s. Then, the specimens were immersed in AS, which was changed every 2 days [4]. Seven days later, specimens were stored in AS for remineralization at 37 °C for another 7 days [14].

A nanoindenter (Hysitron Triboscope, Minnesota, USA) equipped with a conical liquid diamond indent tip (nominal radius of ~1 μm) was used for the scratch tests. Scratch tests were conducted on wet enamel surface under constant load of 1000 μN. The length of the scratch was 10 μm; the sliding speed was 0.5 μm/s. Ten scratches were made on each sample, and the distance between them was at least 10 μm. During each scratch test, normal load, normal displacement, and lateral force were continuously recorded.

**Fig. 2:**
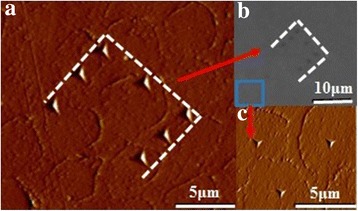
Marked points of nanoindentation. **a** Statistical parametric mapping (SPM) of marked points. **b** Optical microscope (OM) of marked points. **c** Test points in SPM

The same device equipped with a Berkovich tip (Hysitron Triboscope, Minnesota, USA) was used to measure the nanohardness and elastic modulus of the samples. To ensure the tests were made at almost the same position before and after bleaching, points with a maximum load of 9000 μN were marked on the samples (Fig. 2). Nanoindentation test was performed around the marked points, and the distance between them was at least 5 μm (Fig. 2). A maximum load of 2000 μN was set for all nanoindentation tests, and the loading and unloading time was 10 s. Ten indentations were done on each wet enamel surface.

The nanoscratch and indentation tests were conducted before bleaching (baseline) immediately after bleaching and after remineralization for 7 days as shown in Fig. 1.

After bleaching, two specimens from each group were investigated using field emission gun scanning electron microscope (SEM, INSPECT F, Czech Republic).

Statistical analyses were performed by SPSS 16.0. Tukey’s test was used to evaluate mean nanohardness (*H*) and elastic modulus (*E*) of each group between two time intervals (between baseline and after bleaching and between after bleaching and after remineralization).

The values of hardness changes (HC) and elastic modulus changes (EC) of each specimen were calculated according to the following formula: HC_1_ represents the percentage change of nanohardness between baseline and after bleaching:

$$ {\mathrm{HC}}_1 = \left({H}_{\mathrm{bleaching}}-{H}_{\mathrm{baseline}}\right)/{H}_{\mathrm{baseline}}. $$

HC_2_ represents the percentage change of nanohardness after bleaching and after remineralization:

$$ {\mathrm{HC}}_2=\left({H}_{\mathrm{remineralization}}-{H}_{\mathrm{bleaching}}\right)/\ {H}_{\mathrm{bleaching}}. $$

EC_1_ represents the percentage change in elastic modulus between baseline and after bleaching:

$$ {\mathrm{EC}}_1 = \left({E}_{\mathrm{bleaching}}-{E}_{\mathrm{baseline}}\right)/{E}_{\mathrm{baseline}}. $$

EC_2_ represents the percentage change in elastic modulus between after bleaching and after remineralization:

$$ {\mathrm{EC}}_2=\left({E}_{\mathrm{remineralization}}-{E}_{\mathrm{bleaching}}\right)/\ {E}_{\mathrm{bleaching}}. $$

The ANOVA was used to compare the HC and EC values among the groups. All the levels of significance were established at *α* = 0.05.

## Results and Discussion

**Fig. 3:**
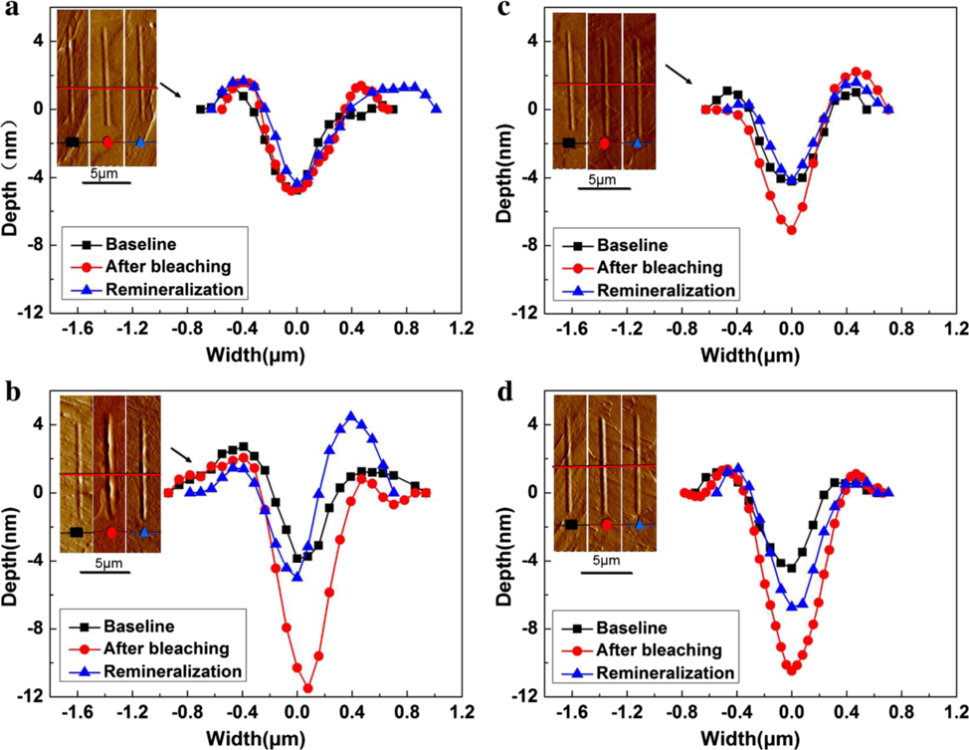
Scratches observed by SPM and the residual traversal profiles of scratch tests. The *small pictures in the left corner* are the scratches observed by SPM. The *red line* shows where the traversal profiles of the scratch tests were got. **a** Control group. **b** 12HP group. **c** 35CP group. **d** 15CP group

The pictures of the scratches observed by SPM and the residual scratch width depths under a constant load of 1000 μN are presented in Fig. 3. The scratch depth of the control groups did not change in the whole process. The pile up on the edges of the grooves represented the plastic deformation undergone by the enamel. For the three bleaching groups, the scratch depth increased after bleaching: 12HP > 15CP > 35CP. And 7 days after bleaching, the scratch depth decreased but was still deeper than baseline.

**Fig. 4:**
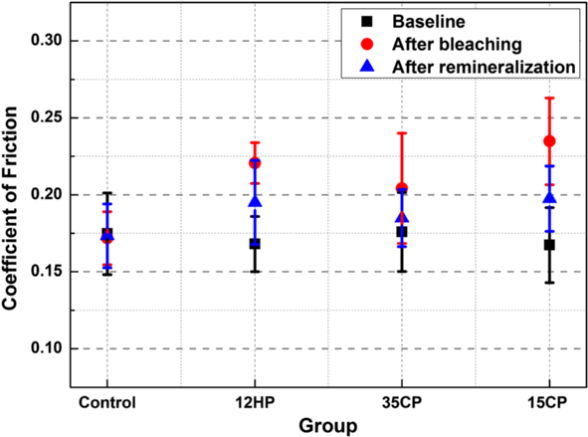
The coefficient of friction changes of each group

Figure 4 shows the coefficient of friction (COF) of the different groups obtained during scratching. The COF for control treatment did not change in the three interval times. The COF of the three bleaching groups increased after bleaching: 15CP > 12HP > 35CP. While 7 days after bleaching, the COF decreased but was still higher than baseline. The change tendency of COF was almost the same as that of the scratch depth changes.

**Fig 5:**
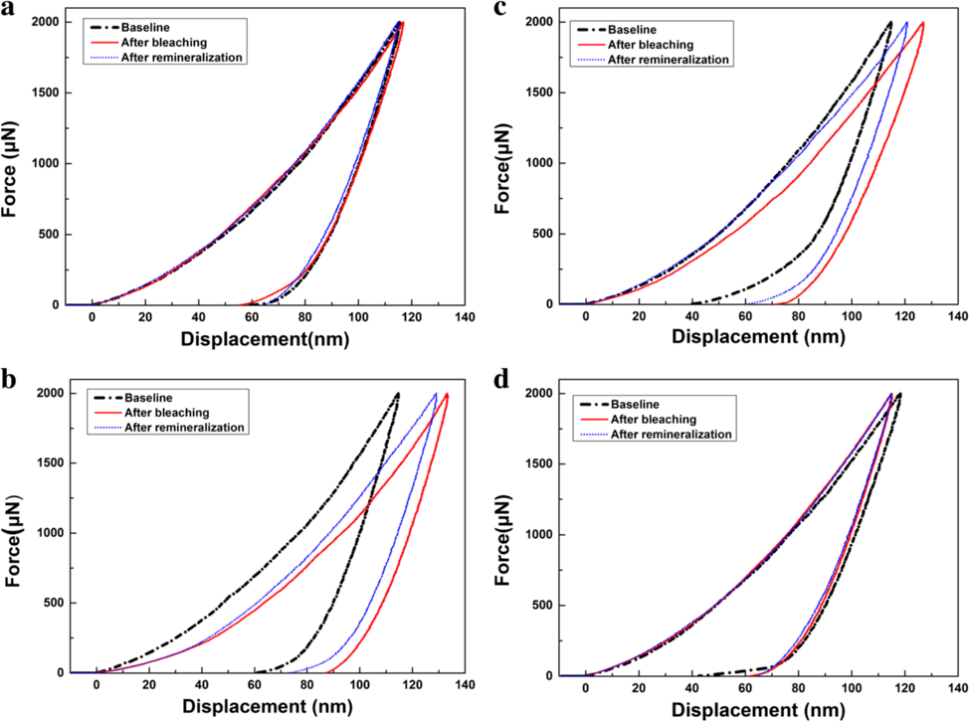
Characteristic load-displacement curves of each group. **a** Control group. **b** 12HP group. **c** 15CP group. **d** 35CP group

Figure 5 is the characteristics load-displacement curves of each group. The characteristic curves proved that at the load of 2000 μN, the indention depth for all the groups was no more than 140 nm. For the control group, the indention depth remained unchanged in the experiment; for 12HP group and 15CP group, the indention depth increased after bleaching, but it decreased after remineralization; for 35CP group, the indention depth decreased after bleaching and remained unchanged after remineralization.

**Fig. 6:**
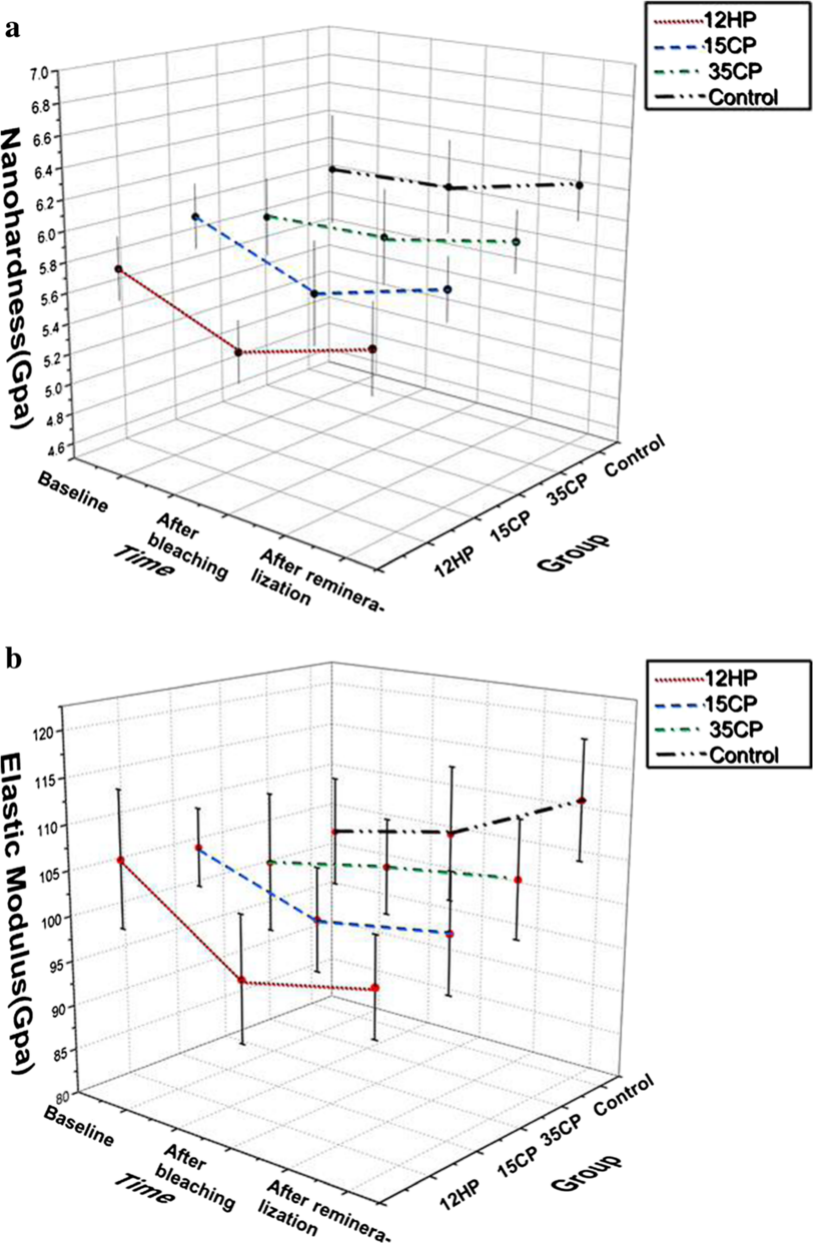
Nanohardness and elastic modulus values of each group in the experiment. **a** Nanohardness values. **b** Elastic modulus values

**Fig. 7:**
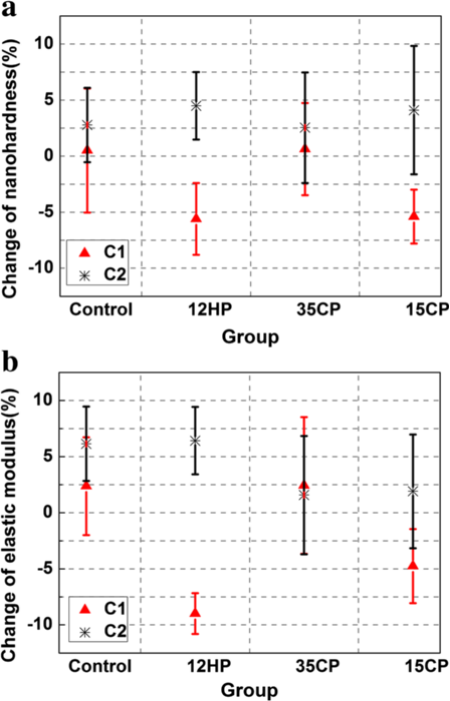
Alternations of mechanical properties between two time intervals. **a** Change of nanohardness. **b** Change of elastic modulus (C_1_ represents the change between after bleaching and baseline; C_2_ represents the change between after remineralization and after bleaching)

Figures 6 and 7 show the nanomechanical property changes in the experiment. The HC_1_ and EC_1_ values of the control group and 35CP group were positive, and those of the 12HP group and 15CP group were negative. All groups showed positive HC_2_ and EC_2_ values. The ANOVA revealed statistically significant difference (*p* < 0.05) for HC_1_ and EC_1_ and no statistical difference (*p* > 0.05) for HC_2_ and EC_2_. HC_1_ values of the 12HP group and 15CP group were almost the same, and they were lower than that of the other two groups. For EC_1_, that of 12HP was the lowest, followed by the 15CP group, and the control group showed no alternation.

Tukey’s test revealed that there were no significant differences (*p* > 0.05) for the change in nanohardness for the control group and 35CP group in the experiment, while that for the 12HP group and 15CP group were significant (*p* < 0.05) after bleaching, but there was no significant (*p* > 0.05) increase in nanohardness after the remineralization.

For the average elastic modulus of the 12HP group, it decreased 9.0 % (*p* < 0.05) after bleaching, followed by the 15CP group which decreased 4.7 % (*p* < 0.05). That of the 35CP group was not statistically significant (*p* > 0.05). After the remineralization, no significant (p > 0.05) change in elastic modulus of all groups was observed.

**Fig. 8:**
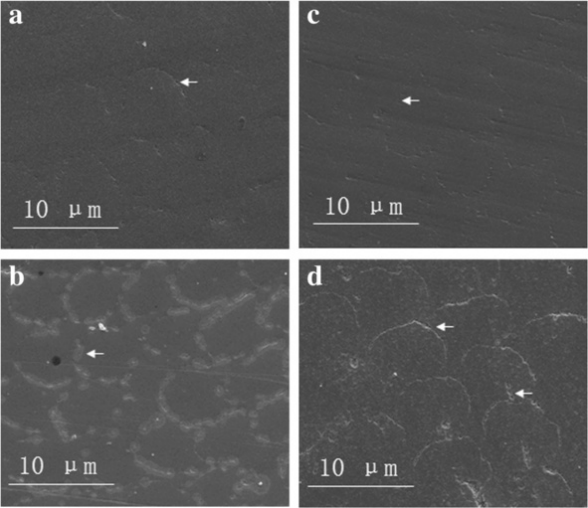
SEM micrographs of tooth enamel after the bleaching procedure. Each panel displays a representative sample from each group. **a** Control group. **b** 12HP group. **c** 35CP group. **d** 15CP group (original magnification at ×10,000)

Representative SEM photographs of bleached specimens are shown in Fig. 8. SEM of the untreated specimens (Fig. 8a) shows a flat enamel surface with some unclear rod structure (white arrow). After the application of the bleaching product containing 12% HP (Fig. 8b), some slight pitting and minimal loss of rod sheath were observed. Regarding the 35CP group, the teeth surfaces appeared quite smooth, and the rod structure was clear and resembled that of the control group (Fig. 8c). For the 15CP group (Fig. 8d), the enamel surfaces showed strongly eroded appearances with voids and loss of material (white arrows) over the surface.

Till now, no research reported how bleaching affected the nanotribological and nanomechanical properties of teeth in wet environment; this is what we want to find out in this research. Also, seldom research reported how the nanotribological and nanomechanical properties changed after remineralization, not to mention in wet condition imitating the oral environment.

As mentioned above, only two researches were found investigating the tribological property changes of bleached tooth [2, 9]. One was done under a dry medium instead of a wet medium [2], while the medium is an important impact factor of the tooth’s nanotribological properties [10]. Previous studies had shown that under a liquid medium, the wear-resistant ability of the teeth is better than that of a dry medium [10]. The other study was on the macrolevel [9], containing only one kind of bleaching product, so in our research we expanded to three commonly used bleaching products to instruct clinic practice.

The results of the nanoscratch tests in the research proved that the bleaching technique reduced the wear resistance of teeth, making the surface layers of enamel became easier to wear off. The three bleaching groups showed an increase in COF and scratch depth after bleaching, especially the 12HP and 15CP groups, which represented a reduction of wear resistance properties. But after storing in AS for 7 days, the COF and scratch depth decreased, representing partly recovery of nanotribological properties. The reason for the result can be explained by the SEM micrographs; the enamel rod was seriously corroded and the sheath showed a demineralization appearance, which also resulted in a decrease of nanohardness and elastic modulus.

One research has shown that the influenced depth resulted from bleaching was less than 20 μm [13], so in the research, we chose the nanoindenter with the test load of 2000 μN to measure the nanomechanical property changes of teeth in order to avoid the interference of influenced depth on the results, which can be proved by the characteristic load-displacement curves (Fig. 5).

**Fig. 9:**
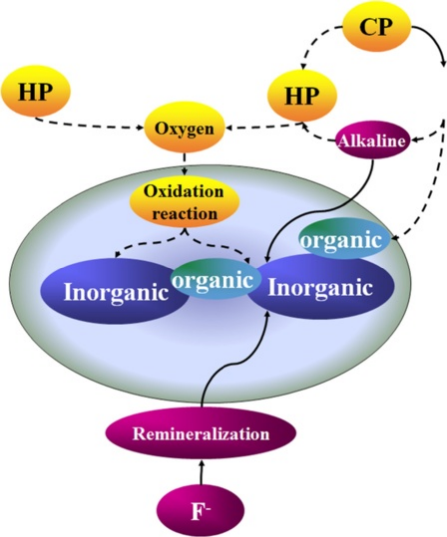
The effects of HP, urea, and F on the changes of nanotribological and nanomechanical properties

The reason for the decrease of nanohardness of the 12HP and 15CP groups may be that HP directly reacted with the inorganic or organic substances of teeth, affecting its glue effect [2]. All these resulted in a loss of inorganic substances, which the nanohardness of tooth enamel is closely related to [15]; hence, the nanohardness of the 12HP and 15CP groups decreased (Fig. 9).

Comparing the results of the 12HP and 35CP groups, we found that the nanohardness of the former decreased about 5.6 % after bleaching, while that of the latter remained almost unchanged (from 5.82 to 5.86 GPa). In fact, CP could decompose into 1/3 HP [16, 17]. So eventually, the concentration of HP is almost the same between the 12HP and 35CP groups. However, the changes in nanomechanical properties were entirely different. The 12HP group showed a decrease in nanohardness and elastic modulus while the 35CP group showed virtually no change. The possible explanations for the phenomenon are as follows: first, the whitening gel of the 35CP group contained 0.11 wt% fluorine (F) [18]. The effective concentration of F required to promote remineralization is 0.1 wt% [19], so the amount of F contained in the bleaching products was enough to promote the remineralization of the teeth and consequently compensated for the decrease in hardness resulting from bleaching [20]. Second, the decomposition of CP could generate not only HP but also alkaline urea [21], which could improve the pH value of the products [17]. Enamel may dissolve in an environment of low pH value [22, 23]; then losing of mineral element happened; and hence the nanomechanical properties were affected. When the pH value increased, the mineral dissolution reduced; then the nanomechanical properties were less affected (Fig. 9).

For the bleaching products containing the same active agent, the change of nanomechanical properties could be different if the concentration and bleaching time were different. For the 15CP group, the bleaching time was 6 h per day, which was much longer than 30 min for the 35CP group. The complete remineralization time of teeth was 24 h in saliva [24]. The remineralization time of the 15CP group was much shorter and could hardly compensate for the decreased nanohardness of the tooth in the process of bleaching, so the nanohardness decreased [25].

The changes in nanomechanical properties were consistent with the changes in anti-wear resistance for 12HP and 15CP groups. The 35CP group showed an increase in COF and scratch depth, but there was virtually no change in nanohardness and elastic modulus in the process. Nanotribological behavior was related, but not determinant to nanomechanical behavior for tooth enamel [26, 27].

These results revealed that the bleaching products containing 12% HP and 15% CP used in the study affected the nanotribological and nanomechanical properties of teeth, but the effects on nanotribological properties can be alleviated after remineralization. The bleaching product containing 35% CP used in the experiment affected the nanotribological properties of teeth but had no effect on the nanomechanical properties of teeth.

All the results were got in vitro, while *in vivo* individual difference exits; oral environment is different from experiment environment, and not everyone would obey the instructions provided by doctors or instructors, so different results may be observed. Further *in vivo* experiments are needed to confirm our results.

## Conclusions

1. The bleaching products containing 12% HP and 15% CP showed corroded teeth surface, while those containing 35% CP had almost no influence on teeth surface appearance in the research.
2. The nanotribological and nanomechanical properties of the 12HP and 15CP groups were affected by bleaching. The nanotribological properties of the 35CP group were affected while the nanomechanical properties were not affected by bleaching.
3. After remineralization, the nanotribological properties got partly recovered, but the nanomechanical properties showed no changes for the three bleaching groups.

## Abbreviations

AS: artificial saliva
COF: coefficient of friction
CP: carbamide peroxide
EC: elastic modulus changes
HBSS: Hank’s balanced salt solution
HC: hardness changes
HP: hydrogen peroxide
SEM: scanning electron microscopy
SPM: statistical parametric mapping
